# Capacitive Bio-Inspired Flow Sensing Cupula

**DOI:** 10.3390/s19112639

**Published:** 2019-06-11

**Authors:** James P. Wissman, Kaushik Sampath, Simon E. Freeman, Charles A. Rohde

**Affiliations:** 1 U.S. Naval Research Laboratory, Code 7165, Washington, DC 20375, USA; kaushik.sampath.ctr.in@nrl.navy.mil (K.S.); charles.rohde@nrl.navy.mil (C.A.R.); 2 U.S. Naval Undersea Warfare Center, Newport, RI 02841, USA; simon.freeman@navy.mil

**Keywords:** capacitive sensing, flow sensing, cupula, liquid metal, stretchable electronics

## Abstract

Submersible robotics have improved in efficiency and versatility by incorporating features found in aquatic life, ranging from thunniform kinematics to shark skin textures. To fully realize these benefits, sensor systems must be incorporated to aid in object detection and navigation through complex flows. Again, inspiration can be taken from biology, drawing on the lateral line sensor systems and neuromast structures found on fish. To maintain a truly soft-bodied robot, a man-made flow sensor must be developed that is entirely complaint, introducing no rigidity to the artificial “skin.” We present a capacitive cupula inspired by superficial neuromasts. Fabricated via lost wax methods and vacuum injection, our 5 mm tall device exhibits a sensitivity of 0.5 pF/mm (capacitance versus tip deflection) and consists of room temperature liquid metal plates embedded in a soft silicone body. In contrast to existing capacitive examples, our sensor incorporates the transducers into the cupula itself rather than at its base. We present a kinematic theory and energy-based approach to approximate capacitance versus flow, resulting in equations that are verified with a combination of experiments and COMSOL simulations.

## 1. Introduction

Recent work in bio-mimetics has shown the advantages taking inspiration from fish for the purposes of creating aquatic robots [1,2]. In particular, body shape [1], thunniform swimming techniques [1,3], and skin texture [4] have been shown to improve efficiency and increase maneuverability. However, behavior [5] is also a key factor in leveraging these advantages. The ability for fish to sense and react to flow enables them to orient efficiently (rheotaxis) [6], detect objects [7], and navigate turbulent flow more efficiently [8]. This concept has been applied to some robots by employing rigid commercial pressure sensors [9,10], but truly biomimetic robots require sensors that are compliant, introducing no rigidity to an already soft bio-mimetic robot. In this work, we develop a capacitive device inspired by superficial cupula structures in fish. In an effort to remain more robust and bio-mimetic, the sensor is created as an all-soft-matter device, consisting of soft silicone rubber and liquid metal. The fabrication methods are unique to this application, employing sacrificial lost wax methods and vacuum filling of channels.

The biological inspiration for this work is derived from the lateral line sensing networks located on fish. Sensors are positioned both on the creature’s surface—called superficial neuromasts—and in recessed channels—called canal neuromasts [11,12]. These two subsystems are considered to have different sensing purposes, such as velocity for superficial versus acceleration sensing for canal (though this is a simplification that ignores factors such as resonance [12]). Our sensor format is most similar to the superficial neuromast, which tends to bend under flow rather than slide like a rigid body as in the case of canal neuromasts [11].

**Figure 1:**
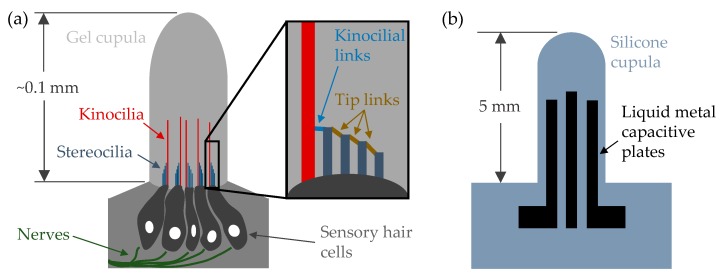
Natural neuromast versus the presented artificial flow sensor. Not drawn to scale, but with general cupula heights indicated. (**a**) a simplified cartoon of the structure of a typical superficial neuromast based on images and descriptions found in the literature [11,13,14]. Inset—detailed image of the hair bundle, highlighting the fiber links between cilia; (**b**) artificial capacitive flow sensor and components.

The biological sensor itself consists of several sensory hair cells that extend microscopic hair (cilia) bundles into the base of a gel cupula [11,13], as shown in Figure 1. These bundles usually include one tall kinocilium and a “staircase” of shorter stereocilia. The cilia are attached to one another with filament “links.” Of particular interest are the kinocilial link and the tip links, as shown in the inset of Figure 1. Under flow, the structure deforms and an electrochemical reaction is produced by relative motion between cilia [13,15]. Specifically, as the hairs move, the links are slackened or pulled taut, activating ion channels/gates at the connection points. While the stereocilia and tip links [15] exist primarily for activating gates, the kinocilia and kinocilial links [13] as well as the gel cupula structure [16,17] have been shown to increase overall sensitivity.

Numerous biomimetic (e.g., fish-, mammal-, or insect-based) flow sensors have been fabricated, tested, and reported in the literature, typically consisting of an artificial cupula or hair that deforms under the influence of moving water or gas [18]. Transducers beneath or within the structure convert the deformation into signals that can be used to approximate flow conditions. Many devices are constructed of microelectromechanical system (MEMS) materials (silicon, aluminum, etc.) and are fabricated with traditional micromachining/lithography techniques [14,16,19,20,21,22,23]. Other examples consist of soft polymer or gel cupulae/hairs on rigid substrates [24] or use flexible (but inextensible) components or substrates [17,25,26]. Few examples exist that could be considered all-soft [27]. Recent sensor development efforts leverage a wide variety of sensing methods, including piezoresistive [16,19,20,25,27], capacitive [21,22,23,28], piezoelectric [14,16,24], ionic polymer-metal composites (IPMC) [17,29], and optic [30]. Comparisons between the approaches are shown in Table 1 in the Results section.

For this work, resistive and capacitive sensing are particularly attractive due to their prevalence in soft robotics. Soft resistive sensors typically consist of rubber embedded with conductive particles and have been developed with applications in medical monitoring [31], motion tracking [31], bio-inspired unmanned air vehicles [32], and prosthetics [33]. Following Ohm’s law, deformation results in either increased or decreased conductance due to changes in micro and macro geometry. In contrast to traditional, non-hyperelastic strain gauges whose material (i.e., constantan alloy foil) can generally be assumed to maintain constant conductivity, the separation and contact of the filler material within conductive elastomers invalidates this assumption and often results in greater sensitivity. These conductive rubbers, however, are vulnerable to hysteresis, viscoelastic responses, and drift under environmental influences, such as humidity or temperature [31,32,33]. (Note that temperature sensitivity for our device is discussed in Appendix A.) For this reason, low toxicity room temperature liquid alloys (gallium-based) offer a promising alternative and have been widely applied in resistive sensing for robotics [34], sensing skins [35], and motion tracking [36].

A simple capacitive pressure sensor consists of two compliant electrodes separated by a soft dielectric. Deforming the electrode surface area or changing the dielectric thickness results in measurable changes in capacitance. This value can be measured accurately even when the electrodes and interconnects have low and/or inconsistent conductivities. As a result, the resistive hysteresis and drifting behavior of conductive rubbers that make some piezoresistive sensing applications infeasible are not a major limitation for measuring capacitance, allowing the use of such materials in capacitive sensors [31,33]. Regardless, liquid metals have been applied to comb capacitor stretch sensors [37] and grid-based capacitive skins [38,39,40], particularly where viscoelasticity, total loss of conductivity, or spotty performance is a concern (e.g., during hyperelastic stretch). A further benefit over resistive sensing is the ease of multiplexing and the ability to use of grid formations without ghosting when multiple nodes (i.e., multi-touch) are affected (mutual capacitance sensing) [41]. This makes capacitive sensing particularly useful for systems where networks of many sensors may be required.

It should be noted, however, that capacitive sensors are often slower in response time when compared to piezoresistive, likely due to the time constant associated with charging and discharging. We have found that the sampling rates of capacitance to digital converters are typically below 1 kHz, whereas analog to digital converters (which can be used for resistive sensing) exhibit frequencies several orders of magnitude higher. In some cases, it may be appropriate to use circuits from condenser (capacitor) microphones, which rely on impedance changes during vibrations and generally include an impedance converter [42] to output a voltage that can be read with an analog to digital converter. This sort of dynamic sensing approach, while capable of sampling high frequency vibrations, is less useful for the work presented here, where a static deformation during flow is observed.

Given the benefits described above, capacitive sensing was chosen for our bio-mimetic device. In contrast to existing examples [21,22,23,28], the sensing plates are located in the cupula itself rather than at its base. In some regards, this more closely resembles biological structures, which consist of hair/cilia encased by gel cupulas. In nature, hairs perform the mechanoelectrical transducing, whereas capacitive plates perform that function in our artificial version at a much larger scale, as seen in Figure 1a,b. Furthermore, although several existing works exist for bio-inspired capacitive flow sensors, to the best of the authors’ knowledge, only one example (seal whisker-inspired [28]) exists that includes testing in water rather than gas flow. This is likely due to difficulties associated with MEMS devices in aqueous environments, such as fragility and corrosion [43]. Our sensor incorporates liquid metal embedded in silicone, making it robust to water and direct handling.

## 2. Design and Operating Principle

**Figure 2:**
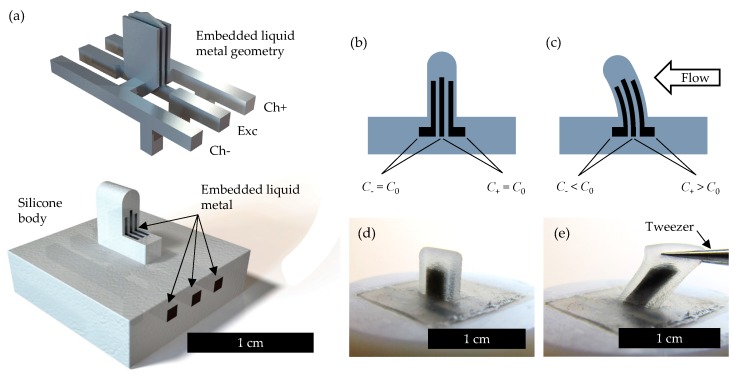
Device overview. (**a**) rendered device image with cupula cutout for clarity (bottom) and a rendering of the internal liquid metal geometry (top). Electrical excitation signals are provided through the channel labeled “Exc” while capacitance is monitored on the receiving ends of “Ch+” (capacitance *C*_+_) and “Ch−” (capacitance *C*_−_); (**b**) the device geometry and behavior under no load. Here, undeformed capacitance values are *C*_+_ = *C*_−_ = *C*_0_; (**c**) the device geometry and behavior under the influence of fluid flow; (**d**) photo of the fabricated device (mounted on a 3D printed holder); (**e**) photo of the cupula being stretched by tweezers to demonstrate its compliance.

The sensor is comprised of three liquid metal plates embedded into the structure of a rubber cupula (Figure 2a). Thus, two parallel plate capacitors are formed, where one central plate, providing the excitation (Exc) for capacitance measurement, is shared for each. In contrast to microtubules [11] that are generally rigid, these capacitive plates are liquid and are not meant to contribute to the stiffness of the structure.

In our device, one capacitor is referred to as the “+” channel (Ch+ with a capacitance of *C*_+_) and one is referred to as the “−” channel (Ch− with a capacitance of *C*_−_). Data are collected based on the difference between the “+” and “−” channels such that, ideally, a sensor under no load should output a capacitance difference value of 0. This is shown in Figure 2b, where the capacitance values are both equal to *C*_0_. When the cupula deflects away from the “+” channel side, the “+” channel increases in capacitance due to stretch while the negative channel decreases due to compression, resulting in a differential increase (Figure 2c). When deflecting away from the “−” channel, the opposite occurs and the capacitance difference decreases. While a single capacitor placed off-center (similar to a unimorph) is adequate for sensing bi-directional flow, a pair of sensing elements (like a bimorph) has the added benefits of increased sensitivity as well as differential removal of noise (limitations are discussed below and in Appendix A). Furthermore, the pair keeps the system mechanically and electrically symmetric, making responses easier to model and characterize.

**Figure 3:**
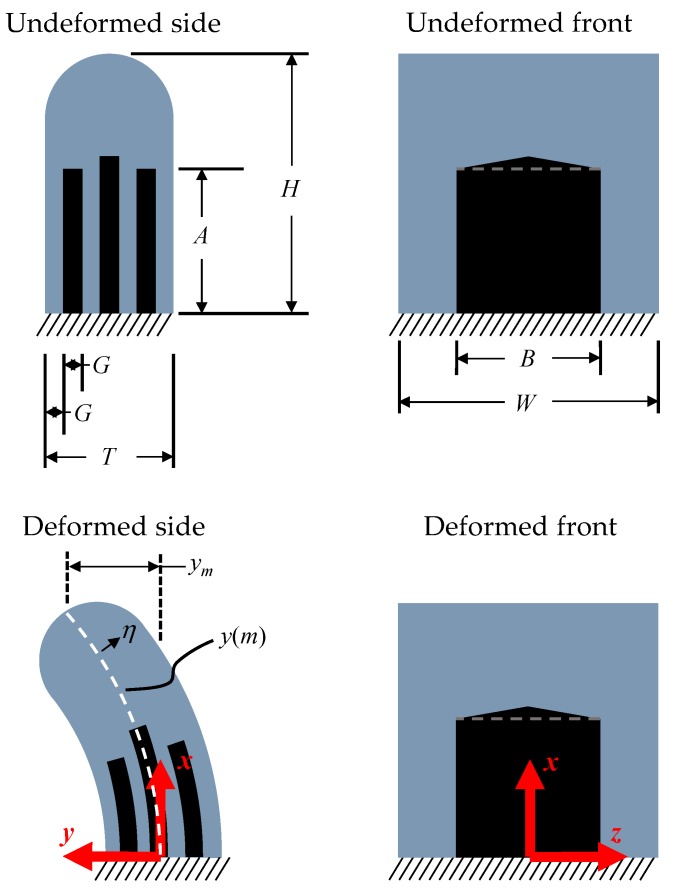
The geometry (not to scale) when undeformed (from the side and the front) as well as the geometry when deformed. Design dimensions: *H* = 5 mm, *W* = 5 mm, *T* = 1.75 mm, *G* = 0.25 mm, *A* = 3 mm, *B* = 3 mm.

Figure 2d shows a photo of the completed device after being mounted to a 3D printed holder. The design dimensions (Figure 3) are 5 mm in height (*H*), 5 mm in width (*W*), and 1.75 mm in thickness (*T*). The liquid metal plates and insulating elastomer are 0.25 mm in thickness (*G*) such that *T* = 7*G*. Each capacitor comprises a 3 mm height (*A*) by 3 mm width (*B*) overlapping area that extends into the cupula. Note that the center plate extends slightly to aid visually during fabrication. This is ignored for the analytic approximation. Furthermore, since the liquid metal is practically incompressible, capable of flowing, and under stress, it realistically can contribute to the mechanics of the system, causing behavior such as buckling. However, for simplicity, the deformation of the liquid metal is assumed to contribute nothing to the system during the analytic calculations—an approximation that is shown to be reasonable by COMSOL agreement.

### 2.1. Kinematics and Capacitance

The behavior of the sensor can be predicted based on kinematics (Figure 3). In classical beam theory, the neutral axis deflection of a cantilever beam under a tip load follows a third order polynomial [44]. While a fourth order polynomial can be used to describe deformation under a uniform distributed load [44] and is more reflective of the cupula in flow, we found that it contributed little to improving accuracy. Furthermore, when compared to the third order approximation, the fourth order increases the complexity of the solutions, which are primarily meant for understanding the mechanics and for scaling. Thus, we approximate that the cupula deflects into a configuration prescribed by

(1) $$y\left(x\right)\approx -\frac{y_{m}}{2H^{3}}x^{3}+\frac{3y_{m}}{2H^{2}}x^{2},\;$$

where *y_m_* is the max deflection, *H* is the cupula height, and *x* is the coordinate along the length (height). This approximation satisfies the essential boundary conditions of zero displacement (*y*(*x*) = 0) and zero slope (*y*′(*x*) = 0) at *x* = 0. Note that we are following a Lagrange notation for derivatives, where *y*′(*x*) is the first derivative with respect to *x* and *y*″(*x*) is the second derivative. A third natural boundary condition is satisfied where the moment is 0 at the free end of the cupula due to lack of traction. This is true when the curvature, which can be approximated as *y*″(*x*) [44] for small deflections, is 0. Thus, we enforce that *y*″(*x*) = 0 at *x* = *H*.

Given the above geometry, the stretch at some distance, *η*, from the neutral axis can be approximated as *λ* ≈ 1 + *ϵ_L_*(*η*). The axial strain along the length of the cupula, can approximated as *ϵ_L_* ≈ *ηy*″(*x*). The average stretch experienced by one capacitor (plate height *A* and dielectric center offset *η* = *G*) can then be calculated as

(2) $$\hat{\lambda }=\frac{1}{A}\int_{0}^{A}\lambda \;dx=1+\frac{3G}{2H^{3}}\left(2H-A\right)y_{m}.$$

Note that the parallel plates’ capacitance is calculated as *C_PP_* = *ϵϵ*_0_*AB*/*G*, where *ϵ*_0_ is the permittivity of free space, *ϵ* is the dielectric constant, *A* is the plate height, *B* is the plate width, and *G* is the dielectric thickness. Under uniaxial loading, the capacitance as a function of stretch can be approximated as *C* ≈ *C*_0_*λ*, where *C*_0_ is the value under no deformation. This approximation assumes that the rubber and liquid metal are incompressible with a Poisson’s ratio of 0.5. Thus, the change in capacitance (for a single capacitor) as a function of max deflection is simply Δ*C* = *C*_0_${\hat{\epsilon }}_{L}$ (where ${\hat{\epsilon }}_{L}$ is the average axial strain experienced by the capacitors), or

(3) $$\Delta C=C_{0}\frac{3G}{2H^{3}}\left(2H-A\right)y_{m}.$$

The sensitivity of Δ*C* to tip deflection, *y_m_*, is doubled for our design due to the presence two differential capacitors. From this theory, we expect that a tip deflection should result in a linear response from the capacitance.

### 2.2. Deformation under Flow

To determine the behavior of the cupula under flow, we take an energy minimization approach that has been valuable in the past for predicting beam deformation [45] and buckled beam snap-through [46]. The system consists of elastic energy from the deforming rubber and work performed by the drag associated water flowing past the structure. The elastic energy [44,46] is a function of the flexural rigidity and the deformation (cupula curvature). In this case, flexural rigidity is the product of the area moment, *I*, and the elastomer’s Young’s modulus, *E*. Thus, the elastic energy is calculated as

(4) $$U_{e}=\int_{0}^{A}\frac{1}{2}I_{1}E\kappa^{2}dx+\int_{A}^{H}\frac{1}{2}I_{2}E\kappa^{2}dx.$$

The energy is calculated with two integrals—one for the lower half of the structure which includes liquid metal and an area moment *I*_1_ and one for the upper half which is solid rubber and has an area moment *I*_2_. The curvature, *κ*, can again be approximated as *y*″(*x*).

We approximate the distributed drag (along the length of the cupula) from the flowing water with the well-known equation *w_D_* = 0.5*C_D_ρWu*^2^ [20,47], where *C_D_* is the drag coefficient (~1.05), *ρ* is the water density, *W* is the cupula width, and *u* is the average flow velocity. The influence of the boundary layer (no-slip condition) is ignored for simplicity, though it is considered in the COMSOL modelling. From here, the total work done by the fluid can be calculated with the integral

(5) $$W_{f}=\int_{0}^{H}w_{D}y\;dx.$$

The use of a constant drag coefficient at Reynold’s numbers below 10,000, which is applicable to our experiments, is a somewhat crude approximation [47,48]. Nevertheless, we apply it in order to acquire a simple closed-form solution.

The total energy of the system, *U* = *U_e_* − *W_f_*, is then described simply as the sum of Equations (4) and (5). At this point, the cupula deformation and the system energy is defined by one free parameter: *y_m_*. The tip deflection for a series of inputs can then be determined by minimizing the energy as a function of *y_m_* (using *dU*/*dy_m_* = 0). Thus, we calculate the change in capacitance as a function of flow for a single capacitor in our device by determining the *y_m_* that minimizes *U* and substituting it into Equation (3):

(6) $$\Delta C_{f}=C_{0}\frac{3C_{D}\rho WH^{2}G\left(2H-A\right)}{32E\left(I_{1}A+I_{2}\left(H-A\right)\right)}u^{2}.$$

Note that this, once again, must be doubled to account for differential capacitors. This theory provides some design variables to follow and indicates that we expect a parabolic response from capacitance as a function of flow velocity.

## 3. Fabrication

A wide variety of liquid metal patterning methods have been proposed and demonstrated in recent years for stretchable electronics [49]. Many techniques, such as stencil lithography [39], laser patterning [50,51], microcontact printing [37], direct write printing [35], and selective wetting [52,53], are primarily 2D in structure. After application on a soft substrate, the circuits can be sealed with layers of elastomer. Some 3D features can be created with 3D printing [54], but more defined structures and higher aspect ratios require freeze printing [55] or freeze casting [56]. These techniques, however, require customized equipment or additional fabrication steps. Instead, we focus on the methods of lost wax mold casting and vacuum injection.

In most examples of casting, elastomer parts are peeled out of reusable molds that can be created with 3D printers [34] or lithography [40]. Multiple molds, sometimes with multiple assembly parts [38], must be used, and several rubber segments may require alignment and bonding to achieve complex geometries [34]. However, overhanging structures and delicate features, as in the case of our proposed design, can be extremely challenging to demold and align. As a result, we employ a sacrificial mold in a lost wax approach. For a single sensor, only one mold is required, though it is dissolved in the fabrication process to release the device.

**Figure 4:**
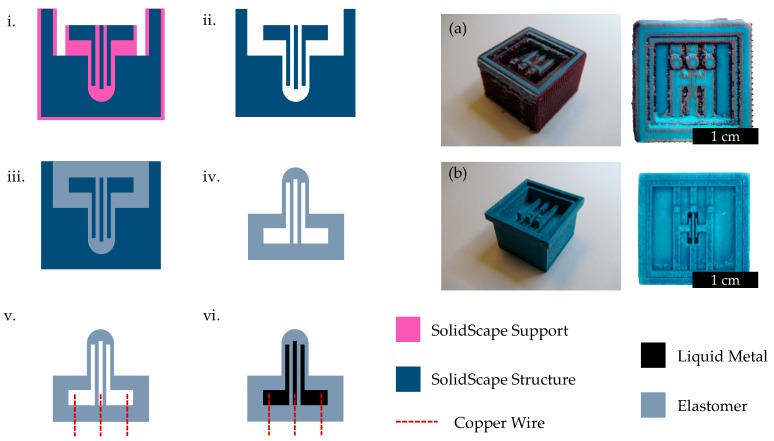
Fabrication flow (not to scale) for creating the flow sensor. i. Complete 3D printed part from SolidScape. Photos of this stage is shown in inset (**a**). ii. Removal of SolidScape Support material. Photos of this stage is shown in inset (**b**) iii. Casting of Ecoflex 0030 elastomer. iv. Removal of SolidScape Structure material. v. Addition of wires and sealing. vi. Vacuum injection of liquid metal and final sealing.

Options for sacrificial lost wax materials include fugitive inks that can be melted [57], poly(lactic acid) that can be vaporized [58], and poly(acrylonitrile-co-butadiene-co-styrene) that can be dissolved in acetone [59]. To avoid custom equipment, excessive temperatures, and harsh solvents, we instead utilize a commercial 3D printer (3Z Pro, SolidScape, Inc., Merrimack, NH, USA) that produces molds from wax-like materials. These materials can melted at low temperatures and dissolved in mild solvents. SolidScape printers and similar components have been applied to the fabrication of scaffolds [60] and microfluidic valves [61,62]. Here, it allows a cupula flow sensor with embedded capacitive sensors and channels for liquid metal wiring to be fabricated in a single casting process. Fabrication steps are shown in Figure 4.

The resolution of the printer (~250 µm) required the device to be of substantial size. In order for a liquid metal parallel plate capacitor of 0.75 mm thickness (including liquid metal plate, dielectric, liquid metal plate) to be substantially deformed, the cupula had to be several millimeters tall. 3D printed parts include both structure and support wax-like materials. The support material was dissolved at a lower temperature of ~60 °C in a bath of non-polar solvent (BIOACT VSO, Vantage Specialty Chemicals, Inc., Gurnee, USA) for several hours. After removing from the bath, heavy drops of BIOACT were soaked up with a Kim Wipe, and the mold was left out overnight to dry.

Before casting elastomer, it is useful to inspect the mold under a microscope to check for debris, particularly between fine features such as the capacitive plates. A strip of paper or a thin wire was used to remove dust and particles as necessary. Next, the uncured polymer can be mixed and casted into the mold. In our case, the silicone elastomer Ecoflex 0030 (Smooth-On, Inc., Macungie, USA) was used for its low Young’s modulus. After degassing in a desiccation chamber until bubbles cease to rise, the samples were left out overnight to cure (higher temperatures increase curing rate but risk deforming or melting the wax-like mold).

The SolidScape structure material is soluble in polar solvents and melts at a higher temperature of about 100 °C. To remove the sensor from the mold, the bulk of the material was softened and scraped away after sitting in an oven set at 100 °C for at least 10 min. The sample was then placed in a small beaker of nearly boiling water until the mold sufficiently melted and dissolved. Isopropyl alcohol was used to clean the surfaces and was injected into the channels with a syringe to remove trapped particles. In many cases, the sample had to be cycled between the hot water bath and alcohol cleaning before all of the mold was removed. The oven set at 100 °C was used to dry the Ecoflex part.

Next, three wires (22 AWG) were stripped on both sides. One end was inserted into the bottom side of the sensor to interface with the liquid metal. The opposite end had a 3-terminal female pin connector soldered for interfacing with external electronics. Sil-Poxy (Smooth-On, Inc.) was used to seal the region between the wire and the Ecoflex. Sil-Poxy was also used to seal the inlets on one side of the sensor, leaving a single inlet for each channel/capacitive plate.

For most liquid metal injection techniques, an inlet and an outlet is required since air must be evacuated [34,38,40], rendering “dead-ends” difficult to successfully fill. One solution to this is to use vacuum filling techniques [63]. We took a similar approach by applying a vacuum with a syringe, and injecting with a second syringe. This is achieved by interfacing between the sensor, the vacuum syringe (30 mL), and the liquid metal syringe (5 mL) with a three way valve. First, the valve was positioned to connect the sensor and the vacuum syringe. Note that a sufficiently large dispensing needle is required to adequately seal with the channel. While pulling a vacuum with the syringe, the valve was rotated to connect the sensor and the liquid metal (eutectic gallium-indium from Sigma-Aldrich, St. Louis, USA) syringe. At this point, the vacuum syringe was released, and liquid metal was carefully injected. Manual injection with a syringe rather than atmospheric pressure, as demonstrated in the literature [63], allows collapsed membranes (common for very soft polymers) to be forced apart by applying additional positive pressure. Cycling this vacuum and injection process can help with the removal of small trapped bubbles.

After injection, the last inlet was sealed with Sil-Poxy and the device was adhered (also with Sil-Poxy) to a 3D-printed (PolyJet, Stratasys, Ltd., Rehovot, Israel) holder to facilitate testing. The interface between the wires and the sensor were sealed with additional Ecoflex 0030 and a cap of epoxy (Devcon 14250, ITW Performance Polymers, Danvers, MA, USA). We found that this sealing was especially important for flow testing, where low pressures during high flow rates within the channel would cause air seep into the device from outside, causing drift and eventual cupula inflation. In some cases, the 3-terminal connectors were removed, re-soldered, and resealed with epoxy to ensure no air could leak through the connector and along the wires.

## 4. Experimental Setups and Methods

To evaluate the performance of the sensor, two experiments were performed. The first examined the response to direct manipulation with an ADMET tensile testing machine (eXpert 2611, ADMET, Inc., Norwood, MA, USA), and the second observed response to water flow in a channel (more details shown in Appendix D). The goal of directly manipulating the cupula was to characterize the capacitive change as a function of cupula tip deflection. While the deformation is not identical to that under liquid flow, this method avoids complexities introduced by vibrations and imprecise flow control, and it is simpler to compare to theory based solely on kinematics. Naturally, the experimentation under flow is a truer evaluation of intended sensor performance.

In every case, capacitance values were recorded with an AD7746 evaluation board in conjunction with an Arduino Due. The AD7746 was used at an update rate of 16.1 Hz and with a measured resolution of about 100 aF, though the chip is capable of 90.9 Hz frequency with lower resolution or 4 aF resolution at lower frequencies. No filtering, aside from that internal to the chip, was applied. While the evaluation board includes means to output to a USB and includes software, using the Arduino Due for communication enabled more accurate timing, including trigger functionality. Data was sent through a serial port and recorded with a MATLAB 2017b script.

### 4.1. Cupula Displacement Testing

For cupula displacement testing, the sensor was mounted to one wall of a box (10 cm × 10 cm × 10 cm), and a notched end effector was attached to the translating portion of the ADMET tensile testing machine. The notch interfaced with the cupula tip. The motion profile featured vertical motions of ±1.25 mm at a quasi-static rate of 0.1 mm/s. This translated to about 1 mm of vertical tip deformation (~2.1% sensor strain). Larger deformations (~1.5 mm vertical deformation and ~3.2% sensor strain) are discussed in the supporting information. To accurately record tip deflection as ground truth, the procedure was recorded by a triggered video camera (Phantom Miro M310, Vision Research, Wayne, PA, USA) whose footage was later analyzed with Tracker Video Analysis and Modeling Tool (copyright © 2018 Douglas Brown), with a resolution of about 20 µm. The Arduino was likewise triggered to begin recording capacitance at the start of the ADMET procedure. The side walls of the box were constructed of acrylic to allow for viewing both when the sensor was in air and when submersed in water, during which the water was connected to ground through a metallic bolt.

### 4.2. Flow Testing

**Figure 5:**
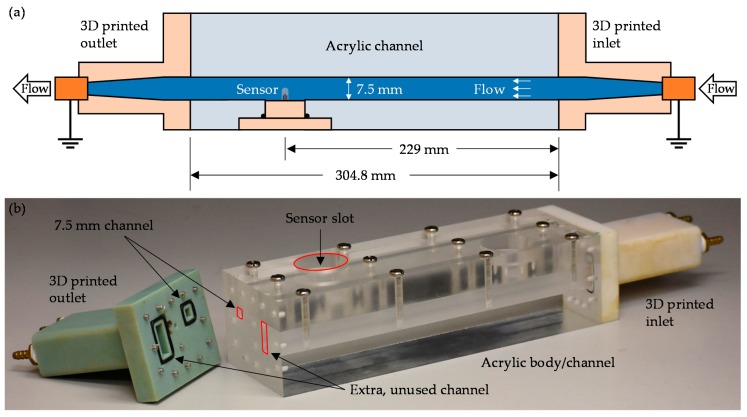
Experimental setup for flow testing. (**a**) schematic of setup (not to scale); (**b**) photo of custom channel with the outlet removed for clarity. Red outlines are added to channels and the used sensor slot for visualization.

For flow testing, a 7.5 mm × 7.5 mm × 304.8 mm channel was constructed out of acrylic (for viewing through the side), including a mount for the sensor to be inserted (see Figure 5) 229 mm downstream. 3D printed flow-transitions on the ends carried flow from brass tube fittings to the channel to prevent separation of the fully developed flow. The brass fittings were grounded (on both ends of the setup) during testing to prevent interference from other electrical sources and charge buildup during flow. A pump (Unilift KP250, Grundfos, Bjerringbro, Denmark) was used to send water from a 6 m × 6 m × 4 m tank through the system. This large tank was used to avoid temperature changes, which can cause sensor drift (see supporting information for more information).

The flow rate was manipulated with an Alicat Liquid Flow Controller (LCR-10LPM-D/5V, Alicat Scientific, Tucson, USA). In particular, rates were increased from 0 L/min to 1 L/min by 0.2 L/min intervals and decreased back to 0 L/min by the same. In practice, a flow rate of 0.01 L/min was used instead of 0 L/min to prevent the controller from entirely shutting its valve—a procedure which resulted in large pressure fluctuations within the channel. No triggering mechanism is included with the flow controller, so capacitive data collection was started manually.

Two scenarios were recorded. For quick sensor evaluation, each interval was set to 20 s, and 5 cycles were completed. This was repeated for the sensor at 0° (its front facing upstream), 45°, 90° (oriented “incorrectly” in the flow), 135°, and 180° (backwards from the 0° case). For experiment at the 0° positioning, video was recorded (manually started) with a camcorder (Vixia HF11, Canon, Inc., Tokyo, Japan) and again analyzed as ground truth with Tracker at a resolution of around 20 µm. Furthermore, a test was performed at 0° with 5 min intervals and 3 cycles to observe longer term behavior.

### 4.3. COMSOL Setup

Finite element flow–structure interaction (FSI) simulations using COMSOL Multiphysics 5.3a are performed to model the displacement of the cupula tip under different flow conditions. These simulations provide a bridge between the experimentally measured tip displacements and the analytical model, enhancing our understanding of the conditions under which both are relevant.

The cross-section of the cupula (5 × 5 mm^2^) is 44% of that of the present flow channel (7.5 × 7.5 mm^2^), resulting in a significant obstruction to the flow profile upon encountering the cupula. The flow is expected to accelerate around the cupula increasing the force experienced by it, and thereby its deformation. In its intended application on an underwater vehicle, this is not expected to occur, as the flow will be unbounded on the outside. The analytic expression derived earlier is closer to the latter, assumes that the deformation is a result of the form drag experienced by the cupula under uniform flow, which is closer to external flow conditions.

**Figure 6:**
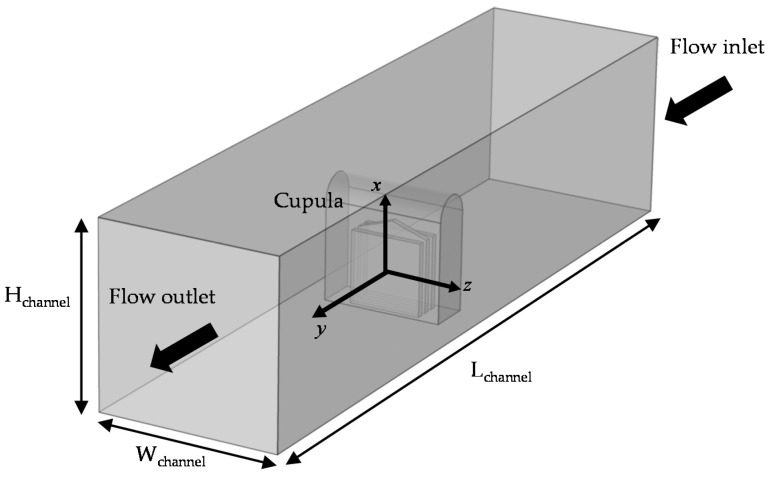
Setup schematic for the finite element fluid-structure interaction simulations performed using COMSOL 5.3a to model the cupula tip displacement under various flow conditions.

Therefore, two simulations are performed, one replicating the present experiments with the same cross-section as the flow channel (denoted as FSI-1) and the other with the externally unbounded flow (denoted as FSI-2), presumably more representative of the analytical solution. The simulation setup is shown in Figure 6. The cupula geometry is matched exactly to that of its fabrication design, i.e., three liquid metal inclusions in an Ecoflex 0030 housing of the same shape. The elastic modulus of the Ecoflex in both simulations is taken to be 125 kPa, based on test measurements and following [64]. The simulation domain (Figure 6) is rectangular with the cupula fixed at its bottom center. The streamwise, wall-normal and spanwise directions are represented by the *y*-, *x*- and *z*-axis respectively. The flow inlet and outlet are marked accordingly and the flow–structure interactions between the cupula and the water flow (outside) as well as liquid metal (inside) are modeled separately. The volumetric flow rates vary from 0.1 to 1 L/min in increments of 0.1 L/min, following the experimental conditions for both the simulations.

The size of the FSI-1 domain is 7.5 × 7.5 × 30 mm^3^, with cross-section matching exactly that of the flow channel. Appropriately, a no-slip flow boundary condition is applied on the side, bottom and top walls of the domain in FSI-1. The inlet flow condition is assumed fully developed and laminar, based on the Reynolds number estimated for the highest flow rate and half-channel height of 1110.

The size of the FSI-2 domain is 11.25 × 11.25 × 30 mm^3^, slightly larger than that of FSI-1 to ensure that the entire wake of the cupula is captured in both dimensions. Owing to the increased size and an absence of any other length scale operating on this ‘external flow’, the inlet flow condition is assumed fully developed and turbulent (k-ω model). A no-slip flow boundary condition is applied on the bottom wall, whereas the side and top boundaries are considered open, representative of an externally unbounded flow as discussed previously.

Both simulations employ a zero-pressure condition for the outflow. A physics based tetrahedral mesh is used, with element sizes ranging from 0.30 to 9.0 mm and 0.45 to 3.0 mm for the FSI-1 and FSI-2 cases, respectively. Furthermore, to evaluate the effect of the elastic modulus on the tip displacement, FSI-1 simulations are also performed for moduli of 60 kPa (a lower value that has also been reported for Ecoflex 0030 [65]) and 1 MPa (an approximation for typical 10:1 polydimethylsiloxane).

## 5. Results and Discussion

The following results are presented from a single sensor, tested both in direct tip deflection and in water flow. Complete data from the single device, including six trials of small tip deflection, nine trials of large tip deflection, and a total of 13 flow trials at varying sensor orientations, are presented in the Appendix B and Appendix C. Here, we present the primary results and outcomes.

In all tests, due to fabrication imperfections and asymmetry in the device, the differential capacitance measured across the device did not fall on 0 pF. Instead, the “+” channel demonstrated a *C*_0_ of ~1.91 pF while the “−” channel showed ~1.131 pF. This was determined by taking the difference between measurements on the sensor and measurements on devices fabricated without capacitive plates extending into the cupula. Note that some error is expected due to differences in parasitic capacitances between devices. Nevertheless, we can extract an approximate dielectric constant for Ecoflex 0030 of about 4.77, which is in reasonable agreement (given geometric tolerances) with literature values that ranges from 2.8 [66] to 4.4 [67] depending on factors such as testing input signal frequency [67].

### 5.1. Capacitance vs. Deflection

**Figure 7:**
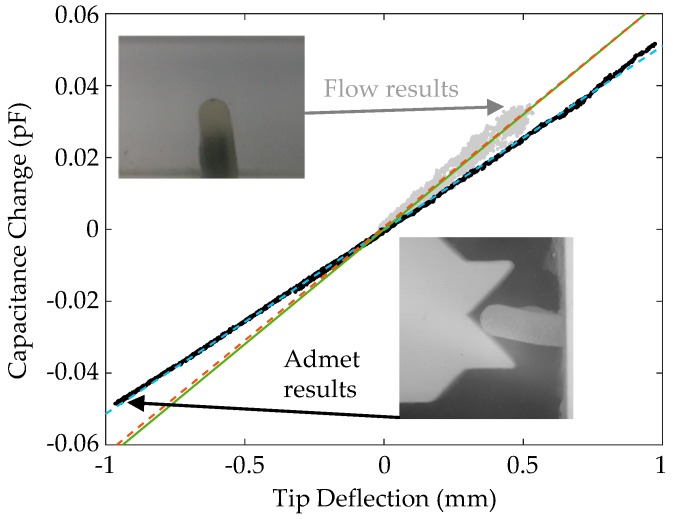
Results of capacitance change versus tip deflection experiments. Black points—data taken from direct manipulation with the ADMET tester (see bottom right inset). Blue dotted line—Fit to ADMET data. Grey points—data taken from flow channel experiments (see top right inset). Red dotted line—fit to flow data; solid green line—theoretical values.

The results from a representative direct displacement test are reported in Figure 7, where capacitive change is plotted as a function of tip deflection while the device was immersed in water. A plot with all six trials’ identical runs is shown in Appendix B. In agreement with the theory, the trend is linear with a sensitivity of about 0.0512 pF/mm. In fact, Equation (3), assuming design dimensions and the average *C*_0_ of 1.521 pF, predicts a sensitivity of 0.0639 pF/mm (falling more in line with flow results). The theoretical value will be off due to an error in the calculation of *C*_0_, as discussed above. However, the theory appears to overestimate sensitivity, likely due to simplifying assumptions on geometry and lack of consideration of mechanical effects such as membrane buckling, which can be seen in the insets of Figure 7.

During ADMET testing, hysteresis varied slightly from test to test. For a tip deflection of 0.4 mm in the data plotted in Figure 7, capacitance change increased by 0.53 fF (~3%) from the upstroke to the downstroke. During another test, the increase was 1.9 fF (~10%). Spread of data points indicates that our sensor can resolve displacements of about 40 µm in terms of accuracy while being able to detect dynamic changes on the order of microns or smaller, though future dynamic testing is required to verify this. Some hysteresis and drift could likely be caused by factors such as viscoelasticity and movement of liquid metal within the device. It should be noted that large deformations (~1.5 mm deflection) resulted in increased nonlinear behavior and increased hysteresis (see Appendix B). This was well beyond what we encountered during flow experiments and requires additional study for further understanding. Capacitive change versus deflection while in air was found to be nearly identical to the aqueous case (see Appendix B).

For a single device, capacitance versus tip deflection sensitivities varied from 0.0511 to 0.0543 pF/mm. In particular, values increased by about 0.003 pF/mm between two separate tip deflection test sessions while varying by less than 0.0005 pF/mm within each. It is likely that handling the device between experimentations resulted in differing overall sensor geometry as the liquid metal and soft elastomer body would settle into various minimum energy states. Trapped air due to imperfect fabrication could also contribute.

### 5.2. Capacitance vs. Flow

Figure 7 also reports point clouds for the relationship between deflection and capacitance during the 0° flow experiment when video was recorded. Here, we see that the sensitivity is 0.0632 pF/mm. Falling closer to the mathematical prediction, this suggests that the flow may have resulted in deformations that more closely followed those prescribed in the theoretical kinematics. However, the fluid–structure interactions are more complex than what was assumed in the theory, and so other factors such as pressure differences on either side of the cupula could be playing a role. The spread of the point clouds is due to several factors. First, the flow controller (and its internal flow sensor) is imperfect, resulting in horizontal spread. Since the flow controller and the bio-inspired sensor could not be perfectly synchronized together, some error due to temporal offset was present in Figure 7. Second, there were vibrations that could not be appropriately resolved without higher sampling rates and proper triggering. Finally, there is some sensor hysteresis and drift, as mentioned above, due to viscoelasticity and liquid metal flow.

**Figure 8:**
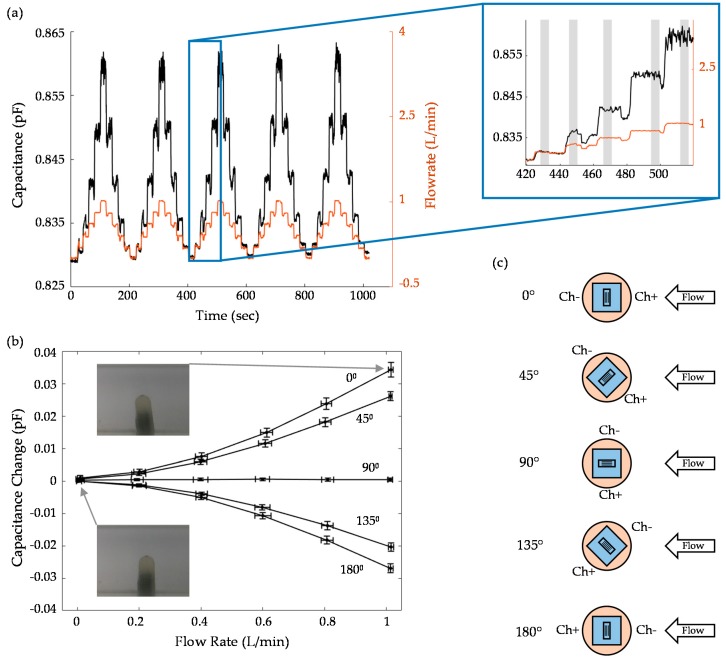
Flow data. (**a**) time dependent capacitance and flow rate during set of testing. The inset shows details of a particular time period; (**b**) averaged sensor results as a function of flow rate; (**c**) an illustration of the different angular orientations whose data is shown in (**b**).

Capacitive output is compared to flow speed as a function of time in Figure 8a, where a 20 s interval testing session is shown. The inset magnifies a particular set of times, highlighting how well the sensor follows the data output from the flow controller. To achieve the capacitive change as a function of flow in Figure 8b, 5-s intervals of data are taken from the middle of each 20 s step. Standard deviation of the flow rate is minimized to avoid large changes during the controller’s output. These 5-s intervals are indicated by the grey boxes in Figure 8a. Figure 8b is an average of several trials (5 for 0° and 2 for the remaining angles), all of which are reported in Appendix C. Sensor orientation was varied as indicated by the angles illustrated in Figure 8c.

The first observation is that the response is nonlinear in shape, as indicated by the theory discussed above, which predicts quadratic. However, assuming an Ecoflex 0030 Young’s modulus of 125 kPa [64], the sensitivity is underestimated by 86% when compared to the 0° average at 1 L/min. This is largely due to the impact of fluid-structure interactions around the cupula, which occupies a large percentage of the channel’s cross-section. During its intended application in a much larger free-stream (unbounded flow), the theory should more accurately predict behavior. To verify this claim, we performed COMSOL simulations, as discussed below.

The asymmetry of the device is again shown in the capacitive change versus flow plot, where 180° does not perfectly mirror 0°. However, we do see the expected behavior, where sensitivity decreases to nearly zero at 90°. In this case, any deformation to the device should nearly equally impact both sides of the device, resulting in a capacitive change of zero. Furthermore, the device is physically less likely to deflect at 90° due to the higher area moment in relation to the fluid flow.

Especially at higher flow rates, large vibrations are apparent in the sensor output due to dynamic flow–structure interactions. Note that the Reynold’s number at 1 L/min is 2300, indicating a transitioning flow for the channel (ignoring the interaction with the sensor). Furthermore, the Strouhal number for a flat plate is 0.15–0.2 [68,69], indicating a vortex shedding frequency of around 12 Hz and below for our system. With the low (16.1 Hz) capacitance sampling rate, a thorough study of vibrations was not in the scope of this study. Instead, these features simply contribute to the vertical error bars in Figure 8b. However, with the AD7746 (at a max frequency of 90.9 Hz), frequency studies are feasible and of interest for future work.

Drift during flow testing was best reflected by the long-term (~3.5 h) test, where 0 L/min values increased by about 0.7 fF. The data is plotted in Appendix C. Sensitivity did change from test to test. After substantial testing and handling (including squeezing and stretching the sample), the final experiment showed a sensitivity decrease of about 15.4% from previous trials. Note that this final experiment is included in the data presented in Figure 8b and is the flow data plotted in Figure 7 and Figure 8a.

Since the sensor response to flow is nonlinear, it is more useful to look at minimum resolvable flow rather than an overall resolution. For our setup, the initial 0.2 L/min (0.06 m/s within the channel) step was clearly identifiable based on the error bars and the long term testing. This could be described as the threshold accuracy. As with displacement testing, much smaller deviations in flow can be detected, though not accurately classified as a specific rate. This is demonstrated by the Figure 8a inset, which shows sensor changes to very small changes in flowrate.

### 5.3. COMSOL Results

**Figure 9:**
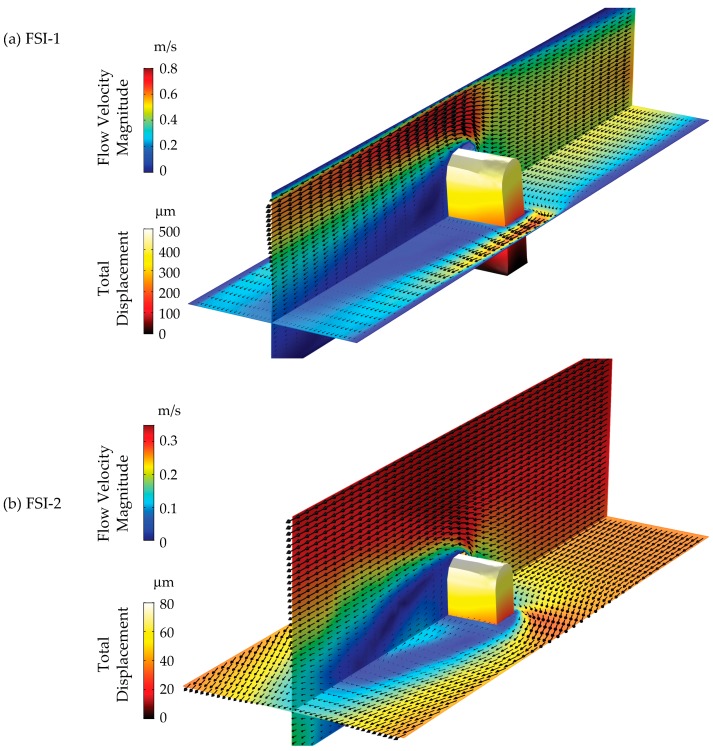
Flow velocity magnitude contour overlaid with velocity vectors on the streamwise-wall-normal and streamwise-spanwise planes in conjunction with surface contours of the total cupula displacement at 1 L/min for (**a**) FSI-1 and (**b**) FSI-2. Note the difference in color-map scales.

Figure 9 shows snapshots of simulation results to elucidate salient features. Contour plots of the flow velocity magnitude overlaid with velocity vectors for the streamwise-wall-normal and streamwise-spanwise planes are shown for (a) FSI-1 and (b) FSI-2. Surface contours of the total displacement of the cupula are also shown. Note the difference between color-map scales. It is evident that the flow and displacements are substantially different from each other. The effects of the walls in FSI-1 are manifested in the form of the accelerated region (~0.7 m/s) on the sides of the cupula in comparison to the mean velocity (~0.3 m/s). This imposes a pressure gradient across the cupula, resulting in a maximum displacement of ~500 μm at the tip. The wake of the cupula extends more than 15 mm downstream. In comparison, the velocity profiles for FSI-2 are uniform. The maximum velocity magnitude is equal to the mean velocity at the inlet (~0.3 m/s), and the wake of the cupula is substantially more homogenized 15 mm downstream. Consequently, this results in a much lower maximum displacement of ~ 80 μm at the cupula tip.

**Figure 10:**
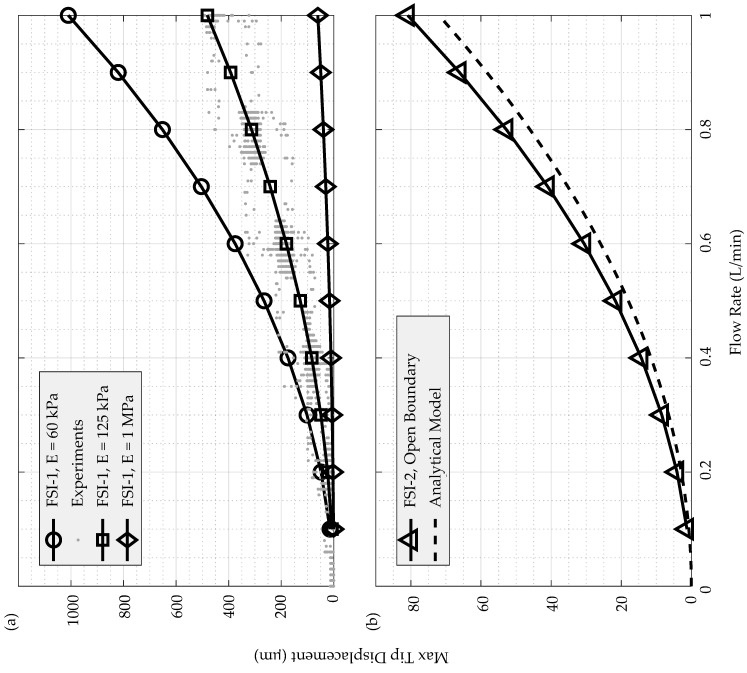
(**a**) maximum tip displacements over the cupula surface versus flowrate for FSI-1 simulations for E = 60 kPa, 125 kPa, and 1 MPa compared with experimental results; (**b**) maximum tip displacements over the cupula surface versus flowrate for FSI-2 simulations compared to the analytical model.

The maximum tip displacements over the cupula surface are plotted w.r.t. the flowrate for FSI-1 in Figure 10a for all the elastic moduli. These are overlaid on a scatter plot of the measured tip displacements in the flow channel. Similarly, Figure 10b displays a comparison between the maximum tip displacements obtained from FSI-2 simulations and those predicted by the analytical model. As evident from Figure 10a, the agreement between the FSI-1 results at E = 125 kPa and the experiments is very good and the deviation of the results from the other two cases confirms that, for Ecoflex 0030, E is closest to 125 kPa. There is also a good agreement between the analytical model and the FSI-2 results. This also confirms the parabolic nature of the displacement predicted by the model. Even though the analytical model does not account for the boundary layer and variations in the velocity profile along the wall-normal direction, it provides a reasonable estimate of the maximum tip displacement. Consequently, the deviation between the model prediction and FSI-2 increases as we move closer to the bottom wall (not shown).

### 5.4. Sensing Threshold Comparison

Table 1 shows how our device compares to several natural and artificial aqueous sensors listed in literature. The parameters include sensing type, cupula material, cupula height, aspect ratio (height over width facing flow) and minimum sensed flow rates demonstrated for both steady (DC) and alternating (AC) flows. Note that minimum values for DC are typically much higher than those for AC. This is likely due to a mix of equipment limitations for steady flows, resonance, and circumvention of drift during alternating flows. When tracking lateral line nerve responses of an African clawed frog, brief flow application with a speaker coil [70] and sinusoidal inputs [71] resulted in detection at velocities around 30 µm/s. However, rheotaxis experiments [6] indicate that, when exposed to a steady flow, some fish do not respond until 5 to 30 mm/s of flow velocity is reached.

**Table 1:** A comparison of natural and artificial aqueous flow sensors. Aspect ratio is height over width facing the flow.

Source	Type	Animal/Cupula Material	Height (mm)	Aspect Ratio	Min DC (mm/s)	Min AC (mm/s)
[70]	Electrochemical	*Xenopus Laevis*	0.1	3	--	0.025 *
[71]	Electrochemical	*Xenopus Laevis*	0.1 [70]	3 [70]	--	0.038
[6]	Electrochemical	*Cheimarrichthys fosteri*	0.036 [72] **	--	5	--
[6]	Electrochemical	*Pagothenia borchgrevinki*	--	--	20	--
[6]	Electrochemical	*Astyanax fasciatus*	0.104 [16]	4 [16]	30	--
[19]	Piezoresistive	SU-8	0.6	7.5	25	0.7
[16]	Piezoresistive	Hydrogel-capped SU-8	0.825	4	--	0.0025
[20]	Piezoresistive	Copper, gold, permalloy	0.82	8.2	200	--
[14]	Piezoelectric	Hydrogel-capped copper	2.7	0.5	75	--
[24]	Piezoelectric	Hydrogel-capped PDMS ***	1.5	1.5	--	0.008
[17]	IPMC ****	PDMS-capped IPMC	35	5	75	--
[30]	Optics	Silicone	3	4.3	70	0.004
[28]	Capacitive	Epoxy	40	20	100	--
This work	Capacitive	Liquid metal, silicone	5	1	60	--

* Based on brief speaker coil input rather than true AC signals. ** Dimension not specified. Only defined as cupula “size.” *** Polydimethylsiloxane. **** Ionic polymer-metal composites.

Likewise, artificial flow sensors demonstrate particularly low thresholds (as low as 2.5 µm/s [16]) for AC signals, whereas the lowest DC flow rates range from 25 mm/s [19] to 100 mm/s [28]. The device sensor presented here falls in the same range of DC values, though future testing is required for a quantitative minimum AC rate. Note that the sensitivity to flow and the minimum threshold can be manipulated using the parameters, such as Young’s modulus, in Equation (7). In terms of size, it is not as large as the only other capacitive example that is demonstrated in water (40 mm “seal whisker” [28]), but it is large compared to many devices (often MEMS-based) that are as small as 700 µm [19]. Regardless, natural cupula, which are often around 100 µm or less in scale [16,70,72], tend to be smaller than the artificial devices.

## 6. Conclusions

We have presented the design, theory, fabrication, and testing of an all-soft-matter bio-inspired flow sensor. Unlike existing capacitive flow sensors, ours includes liquid metal plates embedded within a silicone cupula. The three-dimensional liquid metal paths were created with a combination of lost-wax molding and vacuum injection. Direct cupula manipulation and flow experiments demonstrate repeatable behavior with little hysteresis within single testing sessions and the ability to accurately determine flow rates. The theory does a satisfactory job of predicting capacitance change versus tip deflection and of reflecting flow experiment trends.

Despite the fairly reliable performance within individual sessions, some variability showed up between tests after handling. Given the likely cause of liquid metal flow and deformable elastomer body, a simple design change may decrease deviations. In particular, adding structure, such as a reinforcing grid of supports, within the capacitive plates may force the cupula to remain in a defined geometry rather than allowing multiple minimum energy configurations. Furthermore, the entire structure can be scaled down—a procedure that Equation (7) suggests will linearly decrease sensitivity. This future work will also require new fabrication techniques or a printer with a better resolution, but miniaturization will bring our device closer to examples in nature and will allow us to create a device capable of monitoring boundary layer behavior. Additionally, smaller sensors will enable greater packing density for designing networks.

The theory presented above provides reasonable closed-form equations for evaluating device behavior and guiding future designs. For further understanding of fluid–structure interaction and complex deformations such as membrane buckling, COMSOL finite element simulation was used, verifying the analytic solutions as sound approximations for the physical phenomena. As the device is modified and miniaturized, these models will prove invaluable for optimizing dimensions and materials, particularly as we look at vibrations and possible frequency filtering.

Moving testing methods forward, time-resolved particle image velocimetry (PIV) and capacitance measurements are required for analysis of the cupula under various flow rates. With these capabilities, additional information on turbulence and flow structure can be extracted. Furthermore, vortex-induced vibrations from non-turbulent flow may be tracked, as demonstrated in piezoelectric [14] and IPMC [17] devices in steady flows.

Additional future studies may include exploring the interactions between sensors when placed in a lateral line and testing the response when placed on a deformable substrate. In general, the sensors should be spaced to avoid one device being in the wake of another, which could result in complex behavior. Extrapolating from the COMSOL simulations, the wake is about four cupula body lengths, or 2 cm. Future work might draw from wake interaction studies regarding wind turbines [73]. When placed on a deformable substrate, such as an artificial skin, we expect potentially complex interactions involving changing parasitic capacitances as well as changing flow dynamics. As of now, we cannot make a strong prediction, but it will be a focus as the device finds an application.

The device presented here is uniquely positioned to be applied on a bio-inspired underwater robot. Since the sensor itself includes no rigid components, its application will not reduce the compliance of a robotic “skin.” Additionally, the capacitive design will allow for multiplexing in grid formations for efficient gathering of flow data in a network. With some further design and fabrication refinement, the work presented here will contribute to the navigation and sensing capabilities of swimming robots.

## Figures and Tables

**Figure A1:**
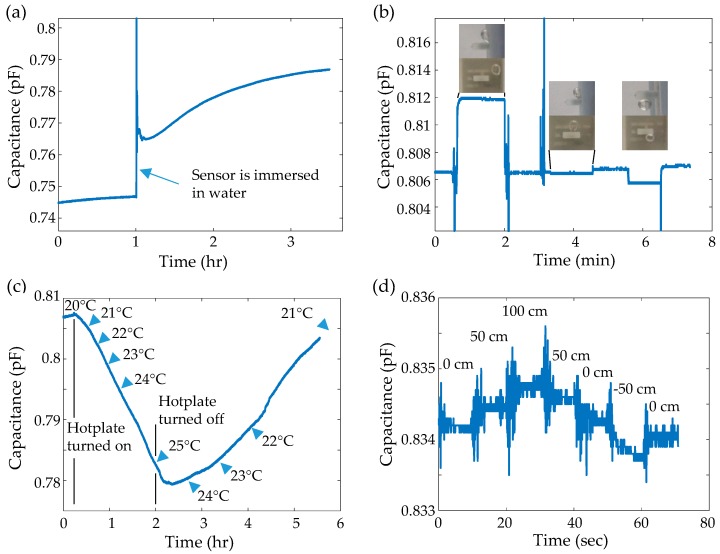
Plots demonstrating various causes of drift. (**a**) drift while sitting in air at 20 °C for one hour, followed by immersion in water for over two ours at 21 °C; (**b**) changes in capacitance due to air bubble placement; (**c**) drift in water due to temperature change; (**d**) capacitance change when lifting and lowering the channel outlet (referenced to sensor height).

**Figure A2:**
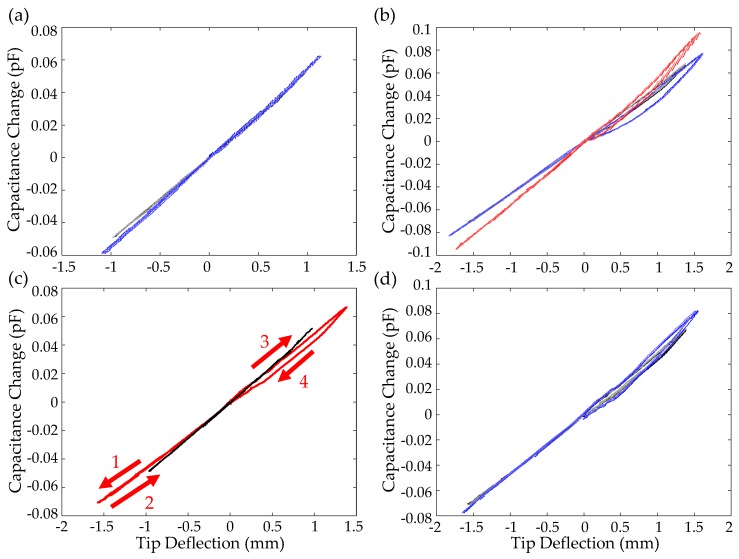
Additional direct deflection data. (**a**) all data for smaller deflections in water. One session of three tests are shades of blue and one set are shades of black; (**b**) all data for larger deflections in water. Three sessions (each of three tests) are in shades of black, blue, and red; (**c**) comparison of a large deflection (red) and small deflection (black). Arrows are included for order of deflection to clarify hysteresis in the larger deflection case; (**d**) comparison of large deflections in air (blue) and in water (black).

**Figure A3:**
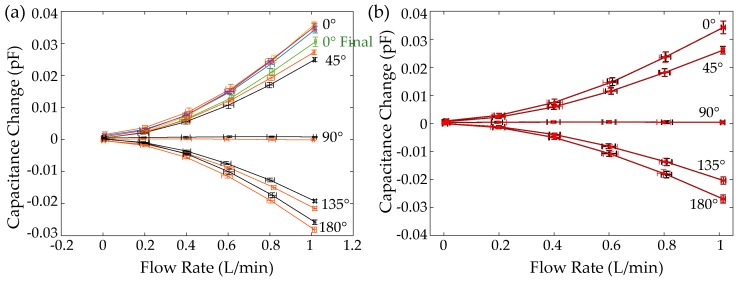
Additional flow data plots. (**a**) individual trials for each angle. The final 0° test is labeled since it was videotaped and was performed after substantial handling; (**b**) comparison of data based on arbitrary 10 s intervals and specified 5-s intervals that lower standard deviation.

**Figure A4:**
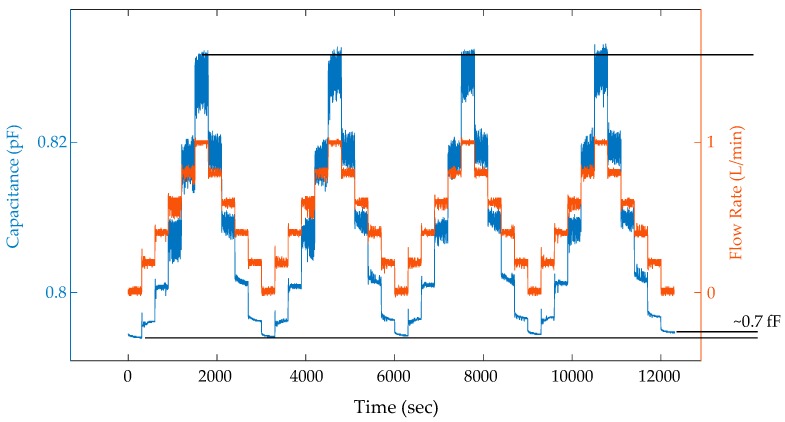
Long-term testing displaying flow rate input and capacitance output over about three hours.

**Figure A5:**
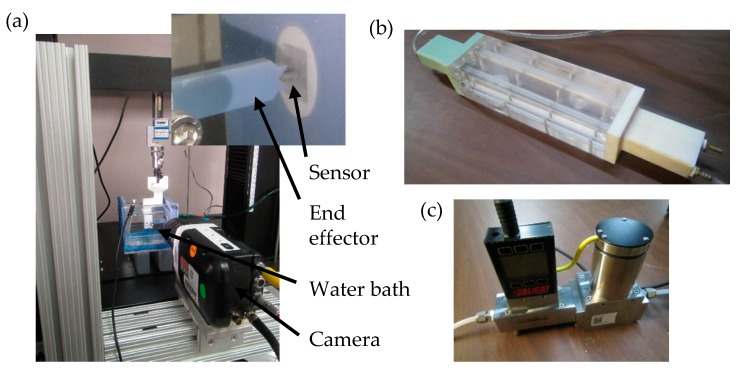
Photos from the experimental setups. (**a**) ADMET test setup with the appropriate portions labeled. The inset shows details for the end effector/sensor interface; (**b**) the custom fabricated flow channel; (**c**) the ALICAT flow controller.

## Appendix A. Sensor Drift

Several outside factors play a role in sensor output. Four major influences are air vs. water environment, air bubbles, temperature, and hydrostatic pressure. Ideally, an undeformed sensor should exhibit no drift. In reality, asymmetries in the device and in the electronics result in capacitance values that can shift either positive or negative, depending on the circumstances. Some examples are represented in Figure A1. The capacitance value generally drifts slowly before settling after several hours (Figure A1a). The immediate change from air to aqueous environment results in an immediate change in capacitance. While buoyancy has an impact since the cupula is horizontal (plates parallel to the ground during the Figure A1a test), we can calculate the associated force when submerged as 0.12 N/m along the cupula length, which would result in a 0.0024 pF change in capacitance according to the theory. Figure A1a, however, displays an increase of nearly 0.02 pF. Furthermore, the capacitance increased regardless of orientation. This indicates that the effect is mostly due to the larger dielectric constant of water. After this initial shift in capacitance, it continues to drift for hours as the elastomer dielectric constant and geometry change, presumably due to water absorption. Bubbles (Figure A1b) can cause changes in capacitance as they deflect the cupula and represent a volume of lower dielectric constant in comparison to surrounding water. In fact, capacitance is used as a method for detecting [74] and measuring [75] bubbles. Temperature (Figure A1c) also results in changing dielectric constants and geometry. Furthermore, trapped bubbles within the liquid metal may expand and contract, causing larger value deviation. The changes in capacitance are at least somewhat reversible. Finally, hydrostatic pressure (Figure A1d) causes a small amount of deviation, limited by the high bulk moduli of silicone and liquid metal. A change of about 150 cm of water in pressure head resulted in about 1 fF of drift.

During experimental testing, steps were taken to avoid the above factors. The sensor generally had at least ~20 min to settle prior to taking data. Bubbles were avoided by using a pump rather than an air pressure pot (which resulted in dissolved gas and bubble formation). The temperature was kept constant by pumping fluid from a large tank of water, and steady hydrostatic pressure was maintained by keeping a fixed channel outlet height.

Note that the above factors also influence the sensor’s sensitivity. However, this has not yet been explored.

## Appendix B. Additional Direct Deflection Data

Capacitance change versus small tip deflection for all six trials (two sessions of three tests—one session in shades of grey and one session in shades of blue) are shown in Figure A2a. One can see that the sets of sessions differ slightly, but trials are very repeatable within each. At larger deflections (Figure A2b, which shows nine trials in three sets of three trials), we see additional hysteresis behavior. One trial includes a decrease (instead of increase as in the lower deflection cases) of 10.8 fF (~24%) from upstroke to downstroke at 1 mm. Nonlinear behavior is expected due to the higher strain values and greater buckling present in such large deformations. However, in some cases (red plot points, for example), one can see steps in the curve. This indicates the possible presence of a stick–slip behavior. Thus, some hysteresis seen here could be due to the testing method rather than the sensor itself. This plot also again demonstrates how the sensor is repeatable within testing sessions. The shades of grey, blue, and red represent three different testing sessions, each with three trials. Prior to the red session, the sensor was handled heavily, evidently impacting the sensitivity.

The plot in Figure A2c demonstrates how a smaller deflection trial (black) compares to a larger deflection trial (red). Furthermore, lines and numbers are included for the larger deflection trial, indicating the direction of motion temporally. Finally, Figure A2d shows a comparison between deflection tests in water (black shades) and in air (blue shades). The difference between the two are negligible, especially compared to possible differences due to device deformation between sessions in general explored.

## Appendix C. Additional Flow Data

Figure A3 shows flow data from individual trials (rather than an overall average). Note that there are two trials for each angle (45°, 90°, 135°, and 180°) in black and red except for 0°, which has 5. The final 0° trial (which was video-taped for position data) is plotted and labeled in green. This trial demonstrates a lower sensitivity, as discussed in the main text, likely due to substantial handling and shifting of the liquid metal/elastomer minimum energy physical configuration.

Average flow data was also compiled based on an arbitrary 10 s interval taken in the middle of each 20 s flow rate step. This is plotted in red on the right plot. The data based on 5-s intervals that minimized flow rate standard deviation is plotted in black. This demonstrates that the capacitance versus flow rate is consistent regardless of the time intervals taken, assuming that it is adequately positioned after transient flow change effects and prior to the next step.

Figure A4 demonstrates how stable the sensor is over about three hours of continuous testing.

## Appendix D. Experimental Setup Images

Figure A5 shows the experimental setups for direct deflection tests and channel flow.
